# *Ferula varia* Trautv. Root Extract: Extraction, Phytochemical Characterization and Evaluation of Biological Activity

**DOI:** 10.3390/molecules31172990

**Published:** 2026-08-26

**Authors:** Marlen K. Smagulov, Yana K. Levaya, Karakoz Zh. Badekova, Assel Sabiyeva, Gulnissa K. Kurmantayeva, Margarita Yu. Ishmuratova, Gülmira Özek, Temel Özek, Izabela Korona-Glowniak, Wirginia Kukula-Koch, Gayane A. Atazhanova

**Affiliations:** 1 Research Park of Biotechnology and Eco-Monitoring, Karaganda Buketov University, Universitetskaya Street, 28, Karaganda 100028, Kazakhstan; marlenkemel@mail.ru (M.K.S.); margarita.ishmur@mail.ru (M.Y.I.); 2 School of Pharmacy, Karaganda Medical University, Gogol Street, 40, Karaganda 100017, Kazakhstan; badekova@qmu.kz (K.Z.B.); sabievaa@qmu.kz (A.S.); kurmantaeva@qmu.kz (G.K.K.); g-atazhanova@mail.ru (G.A.A.); 3 Department of Pharmacognosy, Faculty of Pharmacy, Anadolu University, 26470 Eskisehir, Türkiye; gozek@anadolu.edu.tr (G.Ö.); tozek@anadolu.edu.tr (T.Ö.); 4 Department of Pharmaceutical Microbiology, Medical University of Lublin, 1 Chodzki Street, 20-093 Lublin, Poland; izabela.korona-glowniak@umlub.edu.pl; 5 Department of Pharmacognosy with Medical Plants Garden, Medical University of Lublin, 1 Chodzki Street, 20-093 Lublin, Poland; virginia.kukula@gmail.com

**Keywords:** *Ferula varia*, Apiaceae, HPLC–ESI–QTOF–MS/MS, phytochemical profiling, antioxidant activity, antimicrobial activity, phenolic compounds, sesquiterpene coumarins

## Abstract

**Background/Objectives**: *Ferula varia* Trautv. is a promising medicinal plant of the Apiaceae family and a potential source of biologically active secondary metabolites. The present study aimed to investigate the phytochemical composition and biological activities of *F. varia* root extract. **Methods**: The non-volatile compounds were characterized using high-performance liquid chromatography coupled with mass spectrometry (HPLC–QTOF–ESI-MS/MS) method. Furthermore, the total phenolic and flavonoid contents (TPC and TFC), antioxidant activity, and antimicrobial potential of the extract were evaluated using spectrophotometric and microdilution assays. **Results**: HPLC-ESI-QTOF-MS/MS analysis enabled the tentative identification of 25 metabolites in the *F. varia* root extract, predominantly sesquiterpene coumarins together with organic acids, phenolic acids, coumarins, sesquiterpene esters, and prenylated aromatic compounds. The extract contained 30.06 ± 3.00 mg GAE/g_extr_ of total phenolics and 1.42 ± 0.04 mg RuE/g_extr_ of total flavonoids. Antioxidant assays revealed Trolox equivalent antioxidant capacity (TEAC), total antioxidant capacity (TAC), ferric reducing antioxidant power (FRAP) values of 0.10 ± 0.00 mM, 110.31 ± 4.94 mg AsAE/g_extr_, and 21.95 ± 0.00 mg TE/g_extr_, respectively, while the 2,2-diphenyl-1-picrylhydrazyl (DPPH) assay showed weak radical scavenging activity (IC_50_ > 1 mg/mL). The extract also demonstrated stronger antimicrobial activity against yeasts than against Gram-positive and Gram-negative bacteria, with minimal inhibitory concentration values ranging from 2.5 to 5 mg/mL and 10–20 mg/mL, respectively. **Conclusions**: These findings indicate that *F. varia* roots are a promising source of bioactive compounds exhibiting antioxidant and antimicrobial potential.

## 1. Introduction

Kazakhstan possesses a rich vascular flora of approximately 7000 species. Among these, 278 species are officially designated as medicinal plants according to Order No. 77 of the Ministry of Ecology and Natural Resources (7 March 2023), which established the national list of medicinal plants [1]. Kazakhstan harbors a remarkably rich vascular flora, with at least 1406 medicinal plant species representing 612 genera and 134 families [2]. Owing to its strategic location at the biogeographic interface between Europe and Asia, the country forms an important part of the Central Asian biodiversity hotspot. Its floristic diversity is largely attributed to pronounced habitat heterogeneity and the convergence of contrasting climatic regions, ranging from the cold-temperate zones of Northern Eurasia to the warmer Irano-Turanian region in southern Kazakhstan. These unique ecological and climatic conditions have promoted the development of a highly diverse plant flora with considerable ecological, economic, and ethnomedicinal value. Consequently, Kazakhstan represents an important reservoir of medicinal plant resources and a valuable source of bioactive compounds for pharmacological and phytochemical research [3].

Comprising more than 180 species, the genus *Ferula* L. is a diverse member of the Apiaceae family, widely distributed throughout Central Asia, the Mediterranean region, and the Middle East [4]. In the flora of Kazakhstan, the genus *Ferula* is represented by 51 species, 15 of which are classified as endemic [5,6]. Species of this genus have long been used in traditional medicine owing to their diverse pharmacological properties, including antimicrobial, antioxidant, anti-inflammatory, antidiabetic, and anticancer activities [7,8,9]. These biological effects are mainly associated with the presence of biologically active secondary metabolites such as sesquiterpene coumarins, sulfur-containing compounds, phenolic acids, flavonoids, and essential oils [10]. Numerous representatives of the genus are considered valuable medicinal and aromatic plants and continue to attract increasing scientific interest as promising sources of natural bioactive compounds. Plants of this genus have significant economic value due to their richness in resinous constituents, essential oils, starch, and gums, together with their established uses in traditional medicine and as forage crops [11].

Among them, *Ferula varia* Trautv. is a relatively understudied species despite its occurrence in Central Asian flora and its potential ethnopharmacological importance. Earlier, it has been proposed to evaluate the quality of *F. varia* herb based on the content of the flavonoid cinaroside [12,13]. Previous phytochemical investigations of *Ferula* species have demonstrated that roots are particularly rich in characteristic terpenoid and phenolic constituents, many of which exhibit pronounced biological activities. Traditionally, the root extract of *F. persica* has been used as an antispasmodic, carminative, laxative, and expectorant, as well as for the treatment of diabetes and hypertension [14]. Sesquiterpene esters such as ferutinin, teferdin, and related compounds are regarded as chemotaxonomic markers of several *Ferula* species and are known for their antioxidant, antimicrobial, estrogenic, and cytotoxic properties [15].

Natural antioxidants derived from medicinal plants are currently receiving considerable attention because oxidative stress is closely associated with the development of numerous chronic disorders, including cardiovascular, neurodegenerative, inflammatory, and metabolic diseases [16,17,18]. Plant-derived antioxidants are therefore being actively investigated as safer alternatives to synthetic antioxidants used in the pharmaceutical, cosmetic, and food industries [19,20]. Similarly, the increasing emergence of antibiotic-resistant microorganisms has intensified the search for novel antimicrobial agents of natural origin [21]. Plant extracts and essential oils represent promising sources of such compounds due to their structural diversity and multitarget mechanisms of action.

Although several *Ferula* species have been extensively investigated, *F. varia* remains one of the least explored representatives of the genus, particularly with respect to the phytochemical composition of its underground parts. Previous studies have primarily focused on isolated biological effects or volatile constituents, whereas comprehensive investigations integrating high-resolution metabolite profiling with biological activity assessment are still lacking. Moreover, detailed characterization of the non-volatile metabolome of *F. varia* by UHPLC–QTOF–MS/MS has not been comprehensively reported, leaving significant gaps in the understanding of its chemical diversity and pharmacological potential. Addressing these gaps is essential for identifying bioactive metabolites that may serve as promising candidates for pharmaceutical, nutraceutical, and functional food applications.

The present study represents the first comprehensive HPLC–ESI–QTOF–MS/MS characterization of the secondary metabolites of *F. varia* roots. Several compounds tentatively identified in the extract, including olgoferin, karatavic acid, flavaprenin, ferugin, teferdin, ferutinin, ferusinkin A, badrakemone, karatavicin, and farnesiferone B, have previously been reported from other *Ferula* species [6,7] but are described here for the first time in *F. varia*. These findings substantially expand the known phytochemical profile of the species and further support the remarkable chemical diversity and chemotaxonomic significance of sesquiterpene coumarins within the genus *Ferula*. The study provides a scientific basis for the further investigation of *F. varia* roots as a promising source of natural compounds with antioxidant, antimicrobial, and other pharmacologically relevant activities.

## 2. Results

The results of the phytochemical characterization and biological evaluation of the *F. varia* root extract are presented in this section. The extract was characterized by determining its TFC, TPC and HPLC–ESI–QTOF–MS/MS profiling. Furthermore, its antioxidant capacity, α-amylase inhibitory activity, and antimicrobial activity against Gram-positive bacteria, Gram-negative bacteria, and pathogenic yeasts were evaluated using established in vitro assays.

### 2.1. Phytochemical Analysis of F. varia Root Extract

#### 2.1.1. Quantification of TPC and TFC

The total phenolic and flavonoid contents of the *F. varia* root extract are presented in Table 1. The analysis revealed that the extract contained a relatively high concentration of phenolic compounds, with a TPC value of 30.06 ± 3.00 mg GAE/g_extr_. In contrast, the flavonoid fraction was considerably lower, with a TFC value of 1.42 ± 0.04 mg RuE/g_extr_.

#### 2.1.2. HPLC-ESI-QTOF-MS/MS Characterization

The phytochemical profile of the *F. varia* root extract was characterized by HPLC–ESI–QTOF–MS/MS analysis performed in both negative and positive ionization modes. A total of 25 metabolites were tentatively identified based on their retention times, accurate mass measurements, molecular formulae, mass fragmentation patterns, and comparison with previously reported literature data (Table 2). All compounds were assigned according to MSI identification level 2. A total of 15 metabolites were detected in the negative ionization mode and 12 metabolites in the positive ionization mode.

The annotated constituents included organic acids (citric and succinic acids), phenolic acids (ferulic, chlorogenic, and 3-methoxycinnamic acids), coumarins (bergapten, umbelliferone, 5,8-dihydroxypsoralen, and xanthotoxin), together with numerous sesquiterpene coumarins and related derivatives, including conferdione isomer, olgoferin, karatavic acid, conferone, ferugin, teferdin, ferutinin, ferusinkin A, badrakemone, galbanic acid, omega-acetoxyferprenin, karatavicin, and farnesiferone B. The chromatographic profile of the identified metabolites is presented in Figure 1, while their corresponding MS/MS spectra are provided in the Supplementary File (Figures S1–S27). Chlorogenic acid and umbelliferone were detected in both negative and positive ionization modes, providing complementary MS/MS fragmentation data for its tentative annotation.

Overall, the HPLC–ESI–QTOF–MS/MS analysis demonstrated that the *F. varia* root extract contains a chemically diverse mixture of organic acids, phenolic compounds, coumarins, and sesquiterpene esters, with the latter group representing characteristic constituents of the species.

### 2.2. Evaluation of Biological Activity of the F. varia Root Extract

#### 2.2.1. Antioxidant Activity

The antioxidant potential of the *F. varia* root extract was evaluated using four complementary in vitro assays, namely TEAC, TAC by the phosphomolybdenum method, FRAP, and DPPH radical scavenging activity. The obtained results are summarized in Table 3.

The extract exhibited measurable antioxidant activity in all tested systems. The TEAC assay demonstrated an antioxidant capacity of 0.10 ± 0.00 mM Trolox equivalents, indicating the ability of the extract constituents to neutralize ABTS^•+^ radicals. In the phosphomolybdenum assay, the extract showed a total antioxidant capacity of 110.31 ± 4.94 mg AsAE/g_extr_, reflecting a substantial overall reducing potential. The reducing activity of the extract was further confirmed by the FRAP assay, which yielded a value of 21.95 ± 0.00 mg TE/g_extr_. This result demonstrates the capability of the extract to reduce ferric (Fe^3+^) ions to their ferrous (Fe^2+^) form, suggesting the presence of electron-donating compounds. In contrast, the extract exhibited relatively weak free-radical scavenging activity in the DPPH assay, with an IC_50_ value exceeding 1.0 mg/mL. This finding indicates that relatively high concentrations of the extract were required to achieve 50% scavenging of DPPH radicals.

#### 2.2.2. α-Amylase Inhibitory Activity

The α-Amylase inhibitory activity of the *F. varia* root extract is presented in Table 4. The extract exhibited an inhibitory potential of 6.06 ± 0.55 mg AcrE/g_extr_.

These results indicate the ability of the extract constituents to inhibit α-amylase activity and suggest the presence of compounds with enzyme inhibitory properties.

#### 2.2.3. Antimicrobial and Antifungal Activity

The antimicrobial potential of the *F. varia* root extract was assessed against a range of Gram-positive and Gram-negative bacteria and pathogenic yeast strains based on minimum inhibitory concentration (MIC) and minimum bactericidal/fungicidal concentration (MBC/MFC) values. The results are presented in Table 5. Vancomycin, ciprofloxacin, and nystatin were used as the standard reference drugs (Table 6).

The extract exhibited antimicrobial activity against all tested microorganisms, although the degree of susceptibility varied among the strains. Among Gram-positive bacteria, the strongest inhibitory effect was observed against *M. luteus*, with MIC and MBC values of 5 and 10 mg/mL, respectively. Moderate activity was detected against *B. cereus* (MIC = 10 mg/mL), whereas *S. aureus* ATCC 25923, *S. aureus* ATCC BA1707, *S. epidermidis*, and *E. faecalis* demonstrated lower susceptibility, with MIC values of 20 mg/mL.

Among Gram-negative bacteria, MIC values ranged from 10 to 20 mg/mL. The highest sensitivity was observed for *S.* Typhimurium, *E. coli*, *K. pneumoniae*, and *P. aeruginosa*, each exhibiting MIC values of 10 mg/mL. *P. mirabilis* was comparatively less susceptible, showing both MIC and MBC values of 20 mg/mL. The strongest antimicrobial activity of the extract was observed against yeast strains. *C. glabrata* was the most sensitive microorganism tested, exhibiting MIC and MFC values of 2.5 and 5 mg/mL, respectively. In addition, *C. albicans* and *C. parapsilosis* showed pronounced susceptibility, with MIC and MFC values of 5 mg/mL.

For comparison, the antimicrobial activity of the *F. varia* root extract was evaluated alongside the corresponding reference antimicrobial agents. Vancomycin showed MIC values of 0.12–1.95 mg/L against the tested Gram-positive bacteria, while ciprofloxacin exhibited MIC values of 0.015–0.488 mg/L against Gram-negative bacteria. For the tested yeast strains, nystatin showed MIC values of 0.24–0.48 mg/L. The reference agents demonstrated substantially lower MIC values than the crude *F. varia* extract, confirming their higher antimicrobial potency under the experimental conditions.

Overall, the extract demonstrated stronger antifungal than antibacterial activity. The lowest MIC value recorded in the present study was observed against *C. glabrata* (2.5 mg/mL), indicating that this species was the most susceptible microorganism among all tested strains. In contrast, most bacterial strains required higher inhibitory concentrations (10–20 mg/mL), reflecting a comparatively lower sensitivity to the extract.

## 3. Discussion

The present study offers the first comprehensive characterization of the phytochemical profile and biological activities of *F. varia* root extract. Although several species of the genus *Ferula* have been extensively investigated and the aerial parts of *F. varia* have recently been characterized, detailed studies on the chemical composition and biological activities of the roots remain very limited. To the best of our knowledge, this study represents the first comprehensive HPLC–ESI–QTOF–MS/MS characterization of the secondary metabolite profile of *F. varia* roots. Previously, phytochemical investigations of *F. varia* roots have mainly focused on the isolation and structural elucidation of individual sesquiterpene lactones and their glycosides rather than comprehensive metabolite profiling and, more recently, to the characterization of phenolic constituents in the aerial parts [28]. Suzuki et al. [36] reported several new sesquiterpene lactones from the roots of *F. varia*, whereas Kurimoto et al. [37] subsequently described seven new sesquiterpene lactone glycosides isolated from the methanolic root extract. In contrast, the present study provides a substantially broader phytochemical overview by tentatively identifying 25 metabolites belonging to several structurally distinct classes, thereby considerably expanding the known chemical composition of *F. varia* roots.

Among the identified metabolites, sesquiterpene coumarins are regarded as characteristic chemotaxonomic markers of *Ferula* species and represent one of the largest groups of specialized metabolites within the genus [38,39]. Previous phytochemical investigations have shown that these compounds are widely distributed in the roots, rhizomes, and oleo-gum-resins of numerous *Ferula* species, including *F. communis* [40], *F. assa-foetida* [41,42], *F. huber-morathii* [43], *F. sinkiangensis* [44], *F. latisecta* [45], and *F. flabelliloba* [31]. Although sesquiterpene coumarins are well recognized as characteristic constituents of the genus, several compounds tentatively identified in the present study have not previously been reported from *F. varia*. Olgoferin and karatavic acid have previously been described in *F. persica* [10], conferone, conferdione derivatives, teferdin, ferutinin, and karatavicin were reported from *F. assa-foetida* [41,42], ferusinkin A was isolated from *F. sinkiangensis* [44], whereas farnesiferone B has mainly been reported from *F. assa-foetida* and several Iranian *Ferula* species. Their tentative identification in *F. varia* therefore considerably broadens the known distribution of these metabolites within the genus and highlights the rich chemodiversity of the species.

Their predominance in the present study therefore confirms the characteristic phytochemical pattern of underground organs of *Ferula*. The occurrence of several daucane-type sesquiterpene esters, including ferutinin and teferdin, is particularly noteworthy. These metabolites have repeatedly been reported as major constituents of *Ferula* roots and have attracted considerable attention because of their broad spectrum of biological activities, including antioxidant, antimicrobial, anti-inflammatory, estrogenic, and cytotoxic properties [46]. Ferutinin, in particular, is considered one of the characteristic constituents of several *Ferula* species and has been extensively investigated as a representative bioactive sesquiterpene ester [47].

Another group of detected compounds consisted of phenolic constituents, such as chlorogenic acid, ferulic acid, 3-methoxycinnamic acid, bergapten, umbelliferone, and xanthotoxin. Although these compounds were detected in lower diversity than sesquiterpene coumarins, they represent an important group of metabolites commonly reported in the *Ferula* genus [26]. Notably, chlorogenic acid, ferulic acid, and 3-methoxycinnamic acid have rarely been discussed as constituents of *F. varia* roots. Umbelliferone and furanocoumarins such as bergapten and xanthotoxin are also characteristic constituents of numerous *Ferula* species and have previously been described as taxonomically significant coumarin derivatives within the genus [39].

An interesting observation was the detection of chlorogenic acid in both negative and positive ionization modes. The negative ion mode produced the characteristic fragment ion at *m*/*z* 191, corresponding to quinic acid, together with the diagnostic fragment at *m*/*z* 161, whereas positive ionization generated the protonated molecular ion accompanied by the fragment at *m*/*z* 163. These fragmentation pathways are consistent with previously reported tandem mass spectrometric behavior of chlorogenic acid and are commonly used for its annotation in untargeted metabolomic studies.

The phytochemical profile obtained for the roots differs markedly from that previously reported for the aerial parts of *F. varia*, where phenolic constituents represented the dominant metabolite class [28]. In contrast, the present study demonstrated that the underground organs accumulate a considerably broader spectrum of specialized metabolites, with sesquiterpene coumarins representing the predominant constituents. The simultaneous detection of numerous sesquiterpene coumarins together with phenolic acids, simple coumarins, furanocoumarins, prenylated aromatic compounds, and sesquiterpene esters provides new evidence of the remarkable chemical diversity of *F. varia* roots. These findings substantially expand the phytochemical knowledge of the species and indicate that *F. varia* represents a richer source of bioactive metabolites than previously recognized.

In addition to the qualitative characterization of individual metabolites by HPLC–ESI–QTOF–MS/MS, the quantitative determination of TPC and TFC provides complementary information on the abundance of the major classes of antioxidant constituents. Compared with our previous study [28] on the aerial parts of *F. varia*, the root extract contained a higher level of total phenolics (30.06 ± 3.00 vs. 23.22 ± 1.02 mg GAE/g_extr_) but a lower flavonoid content (1.42 ± 0.04 vs. 2.83 ± 0.98 mg RUE/g_extr_), suggesting organ-specific accumulation of phenolic metabolites. TPC and TFC results are consistent with the HPLC–ESI–QTOF–MS/MS, which revealed the presence of. several phenolic acids, while flavonoids, if present, are likely to occur only in minor amounts. Nouioura et al. [48] showed that *F. communis* ethanol and methanol fruit extracts exhibited the highest TPC values of 62.20 ± 0.11 and 60.82 ± 0.32 mg GAE/g dry weight (DW), respectively, followed by the aqueous extract (44.04 ± 0.22 mg GAE/g DW). Lower TPC values were reported for acetone (19.76 ± 1.19 mg GAE/g DW), ethyl acetate (15.24 ± 0.60 mg GAE/g DW), chloroform (13.94 ± 0.92 mg GAE/g DW), and hexane (6.93 ± 0.43 mg GAE/g DW) extracts. In another study, Tunisian *F. communis* showed markedly higher TPC values in fruits (422 mg GAE/g DW), followed by flowers (207.21 mg GAE/g DW) and stems (129.86 mg GAE/g DW) [24]. A recent study on *F. persica* revealed the highest TPC was observed in the cyclopentyl methyl ether extract (78.3 mg GAE/g), followed by the ethyl acetate (76.3 mg GAE/g) and ethanol (74.5 mg GAE/g) extracts of the aerial parts, whereas root extracts contained 19.5–41.6 mg GAE/g [49].

According to the literature data, chlorogenic acid is one of the major hydroxycinnamic acid derivatives frequently associated with antioxidant activity [50], whereas ferulic acid contributes to the radical scavenging and reducing properties of plant extracts [51]. The observed differences in their content may be reflected in the antioxidant capacity determined by different in vitro assays. The antioxidant activity of *F. varia* root extract was evaluated using assays based on different reaction mechanisms. The extract exhibited moderate antioxidant activity. However, the response varied among the methods. The highest activity was observed in the phosphomolybdenum assay (110.31 mg AsAE/g_extr_), indicating a considerable total reducing capacity. The FRAP assay also demonstrated the ability of the extract to reduce Fe^3+^ ions (21.95 mg TE/g_extr_). In contrast, the DPPH assay showed weak radical scavenging activity (IC_50_ > 1.0 mg/mL). This difference suggests that the extract acts primarily through electron transfer rather than hydrogen atom transfer. Comparable findings have been documented in other *Ferula* species. Hydroethanolic extracts of *F. asafoetida* leaves exhibited stronger DPPH scavenging and ferric reducing activity than gum extracts owing to their higher phenolic and flavonoid contents [52]. The authors concluded that antioxidant capacity positively correlated with total phenolic concentration. In contrast, root extracts enriched in terpenoids and sesquiterpene coumarins often display only moderate DPPH scavenging despite measurable reducing capacity. Several reviews indicate that Ferula species are particularly rich in sesquiterpene coumarins, sulfur-containing metabolites, and terpenoids, compounds more frequently associated with antimicrobial and anti-inflammatory effects than with high radical-scavenging activity [8]. A recent study of *F. communis* reported substantially stronger DPPH activity for leaf extracts IC_50_ value of 0.263 ± 0.008 mg/mL, while methanolic fruit and root extracts exhibited IC_50_ values of 0.076 ± 0.039 mg/mL and 0.820 ± 0.031 mg/mL, respectively [53]. Likewise, antioxidant activity in *F. sinkiangensis* varied considerably among solvent fractions [54]. The ethyl acetate, *n*-butanol, and methanol fractions exhibited DPPH IC_50_ values of 34.1, 33.8, and 40.3 μg/mL, respectively. These values are nearly 25–30-fold lower than the activity observed for *F. varia*, indicating a much higher free-radical scavenging capacity. The authors attributed this effect to the enrichment of phenolic constituents in medium-polarity solvent fractions. The antioxidant activity of *F. varia* was lower than that reported for *F. communis* [48]. Methanolic and ethanolic extracts of *F. communis* exhibited DPPH IC_50_ values of 8.09 and 8.25 μg/mL, respectively, whereas acetone and aqueous extracts showed IC_50_ values of 34.23 and 37.28 μg/mL. In addition, the methanolic extract displayed the highest reducing power (EC_50_ = 17.63 μg) and total antioxidant capacity (72.15 ± 0.11 mg AAE/g). Overall, variations in antioxidant activity among *Ferula* species are largely determined by the extraction solvent, plant organ, and metabolite composition, particularly the abundance of phenolic compounds.

The *F. varia* root extract demonstrated measurable α-amylase inhibitory activity (6.06 ± 0.55 mg AcrE/g_extr_), indicating the presence of constituents capable of suppressing carbohydrate-hydrolyzing enzymes. Recent studies have demonstrated that sesquiterpene coumarins represent a promising class of natural α-amylase inhibitors [55]. Baccari et al. [56] reported that a series of semisynthetic arylidene derivatives obtained from the sesquiterpene coumarin coladonin, isolated from the roots of *F. tunetana*, exhibited potent α-amylase inhibitory activity, with IC_50_ values ranging from 7.24 to 28.98 μM. Ferulic acid identified in the *F. varia* root extract has previously been reported as an effective inhibitor of carbohydrate-hydrolyzing enzymes. Zheng et al. [57] demonstrated that ferulic acid inhibited α-amylase and α-glucosidase with IC_50_ values of 0.622 and 0.866 mg/mL, respectively.

The *F. varia* root extract exhibited antimicrobial activity against a range of Gram-positive and Gram-negative bacteria, as well as yeast strains. Notably, this study represents the first report on the antimicrobial activity of *F. varia* root extract, and the obtained findings were therefore compared with those reported for other *Ferula* species. The 80% methanolic root extract of *F. communis* exhibited greater activity against Gram-positive bacteria, particularly *S. aureus* (MIC = 16.67–18.66 mg/mL), whereas Gram-negative bacteria were less susceptible, with average MIC values exceeding 30 mg/mL [58]. Similar findings were reported for *F. communis* fruit extracts, where the ethanolic extract exhibited the strongest antibacterial activity, with MIC values of 0.312 mg/mL against *E. coli* and 0.625 mg/mL against *B. subtilis* and *P. mirabilis*, while the acetone extract showed MIC values of 2.5 mg/mL against all tested bacterial strains. The superior activity of the ethanolic extract was associated with its higher phenolic and flavonoid contents and greater antioxidant capacity [48]. Likewise, the chloroform extract of the aerial parts of *F. caspica* demonstrated the highest antibacterial activity against *S. aureus* (MIC = 32 μg/mL) and *E. faecalis* (MIC = 64 μg/mL), while *E. coli* and *P. aeruginosa* exhibited markedly lower susceptibility (MIC = 512 μg/mL). The same extract showed only moderate antifungal activity against *Candida* spp., with MIC values ranging from 128 to 256 μg/mL [59]. In contrast, the CO_2_ extract of *F. asafoetida* displayed potent activity against both Gram-positive and Gram-negative bacteria, with MIC values of 7.81 μg/mL for *S. aureus* and 15.63 μg/mL for *E. coli*, *K. pneumoniae*, and *S. enterica*, whereas *C. albicans* was considerably less susceptible (MIC = 62.5 μg/mL) [60].These findings suggest that the antimicrobial activity of *Ferula* species is strongly influenced by both the plant species and the extraction solvent, resulting in different susceptibility patterns among microbial groups. Unlike previously reported extracts, which generally showed greater efficacy against Gram-positive bacteria, the *F. varia* root extract demonstrated comparable or even higher activity against *E. coli*, *S.* Typhimurium, *K. pneumoniae*, and *P. aeruginosa* with MIC = 10 mg/mL, while the strongest antimicrobial effect was observed against *C. glabrata* with MIC = 2.5 mg/mL. For additional comparison, the antimicrobial activity of the *F. varia* root extract was evaluated alongside standard reference agents. Vancomycin showed MIC values of 0.12–1.95 mg/L against the tested Gram-positive bacteria, ciprofloxacin exhibited MIC values of 0.015–0.488 mg/L against Gram-negative bacteria, and nystatin showed MIC values of 0.24–0.48 mg/L against the tested yeast strains. As expected, the reference antimicrobial agents exhibited substantially greater potency than the crude *F. varia* extract. However, direct comparison of the numerical MIC values should be interpreted with caution because the reference drugs are purified antimicrobial compounds, whereas the plant extract represents a complex mixture of constituents and was tested at mg/mL concentrations. Nevertheless, the positive-control results confirm the expected susceptibility of the tested microorganisms and provide an appropriate reference framework for evaluating the antimicrobial activity of the *F. varia* root extract.

The observed antimicrobial activity may be related to the coumarins -rich composition of the extract, as well as phenolic acids. In the present extract, compounds such as ferutinin, umbelliferone, galbanic acid, ferulic and chlorogenic acids have been associated with antimicrobial activity. For example, ferutinin exhibited the strongest antibacterial activity against *S. aureus*, with MIC and MBC values of 25 μM [61]. Shahverdi et al. [62] reported that galbanic acid markedly enhanced the activity of β-lactam antibiotics against *S. aureus*, reducing the MICs of penicillin G and cephalexin by 64-fold and 128-fold, respectively. Borges et al. [63] reported that ferulic acid exhibited broad-spectrum antibacterial activity against *E. coli*, *P. aeruginosa*, *S. aureus*, and *L. monocytogenes*, with MIC values of 100 μg/mL for *E. coli* and *P. aeruginosa* and 1100–1250 μg/mL for Gram-positive bacteria, acting primarily through irreversible disruption of bacterial membrane integrity and increased membrane permeability. Previous studies have demonstrated that chlorogenic acid exhibits broad-spectrum antibacterial activity, with MIC values ranging from 20 to 80 μg/mL [64]. Umbelliferone was reported to exhibit potent antibacterial activity against *B. cereus*, *B. subtilis*, and *K. pneumoniae*, with MIC values of 0.31–0.62 mg/mL [61]. Additionally, umbelliferone showed antifungal activity against both susceptible and multidrug-resistant *C. albicans* strains, with MIC values of 125–175 μg/mL, highlighting its potential as a promising natural antifungal agent [65].

These findings indicate that the biological potential of *F. varia* roots is closely associated with their chemically diverse metabolite profile, particularly the presence of phenolic acids and characteristic sesquiterpene coumarins. Although the extract demonstrated promising antioxidant, enzyme inhibitory, antimicrobial, and antifungal properties in vitro. This study was limited to in vitro assays and tentative metabolite identification. Future investigations should focus on the isolation and quantitative determination of the major constituents, synergistic biological effects, elucidation of their mechanisms of action, and confirmation of efficacy and safety in appropriate in vivo models.

## 4. Materials and Methods

### 4.1. Plant Material

The wild-growing plant *Ferula varia* (Schrenk ex Fisch., C.A.Mey. & Avé-Lall.) Trautv., native to the flora of Kazakhstan, was collected during the flowering period in April 2025 as part of an expedition to the Northern Pre-Balkhash region of the Republic of Kazakhstan. The plant material was collected near the Bektatau Mountains, Aktogai District, Karaganda Region, at 47.74108° N, 74.29520° E. The underground parts of *F. varia* were taxonomically authenticated by botanist Dr. M. Yu. Ishmuratova, and a voucher specimen was deposited in the Herbarium of E.A. Buketov Karaganda University under accession number QAR04328.

### 4.2. Preparation of the Extract

Dried roots (19 g) were subjected to microwave-assisted extraction with 70% aqueous ethanol (*w*/*w*) at a plant material-to-solvent ratio of 1:10 (*w*/*v*) using an ETHOS X advanced microwave extraction system (Milestone, Sorisole, Italy). Extraction was performed at 800 W microwave power for 12 min [66]. The resulting extract was filtered, and the solvent was removed under reduced pressure using an IKA RV 8 rotary evaporator (IKA-Werke GmbH & Co. KG, Breisgau, Germany). The extraction yield was 20.9% (*w*/*w*).

### 4.3. Phytochemical Analysis

#### 4.3.1. Determination of TPC

The TPC of the extracts was determined spectrophotometrically using the Folin–Ciocalteu reagent according to a previously described method [67]. Methanol was used to prepare the extract solutions and gallic acid stock solutions. For the assay, 20 µL of the sample solution, 1560 µL of ultrapure water, and 100 µL of Folin–Ciocalteu reagent were sequentially combined in a 96-deep-well plate using a 12-channel micropipette. Following incubation for 1–8 min, 300 µL of 20% sodium carbonate solution was added, and the mixture was thoroughly mixed. The reaction mixture was then incubated for 2 h at 25 °C in the dark. Subsequently, 300 µL of the reaction mixture was transferred to a 96-well microplate, and absorbance was measured at 760 nm. The TPC was calculated using a five-point calibration curve prepared with gallic acid at concentrations ranging from 0.01 to 1.0 mg/mL. All measurements were performed in triplicate. The gallic acid calibration curve was described by the equation *y* = 0.7897*x* + 0.0606, with an R^2^ value of 0.9915 (Figure S28). The results were expressed as the mean ± standard error and reported as milligrams of gallic acid equivalents per gram of extract (mg GAE/g_extr_).

#### 4.3.2. Determination of TFC

The TFC was quantified using a spectrophotometric aluminum chloride (AlCl_3_) assay based on a previously published procedure [68]. Briefly, 80 µL of either the extract solution or rutin standard was mixed with 80 µL of AlCl_3_ reagent and 1840 µL of absolute ethanol in a 96-deep-well plate. For the blanks, AlCl_3_ was replaced with 10 µL of 99.8% acetic acid. The mixtures were allowed to react for 40 min in the dark, after which 300 µL of each reaction mixture was transferred to a 96-well microplate. Absorbance was subsequently recorded at 415 nm using a microplate reader. Quantification was performed against a seven-point rutin calibration curve covering 0.01–1.0 mg/mL, described by the equation *y* = 1.6177*x* + 0.0479 (R^2^ = 0.9987; Figure S29). The results were calculated as the mean ± standard error and expressed as milligrams of rutin equivalents per gram of extract (mg RuE/g_extr_).

#### 4.3.3. HPLC–ESI–QTOF–MS/MS Analysis

Phytochemical profiling of the extract was conducted using an Agilent 1200 HPLC system coupled with an Agilent 6210 Quadrupole Time-of-Flight (QTOF) mass spectrometer (Agilent Technologies, Santa Clara, CA, USA). The analysis was performed in both negative (ESI−) and positive (ESI+) electrospray ionization mode with a Dual AJS ESI ion source (Agilent Technologies, Santa Clara, CA, USA). Chromatographic separation was achieved using a binary mobile-phase system comprising solvent A (water containing 0.1% formic acid) and solvent B (acetonitrile containing 0.1% formic acid). The gradient program was set as follows: 0–2 min, 99% A/1% B; 2–10 min, a linear change to 80% A/20% B; 10–15 min, 60% A/40% B; 15–17 min, 5% A/95% B; 17–22 min, 5% A/95% B; and 22.1–25 min, restoration of the initial composition (99% A/1% B). The mobile-phase flow rate was 0.2 mL/min, with a total chromatographic run time of 30 min. UV detection was performed at 290 nm. A 5 μL sample volume was injected using the standard injection mode. Mass spectrometric data were acquired in Auto MS/MS mode over an *m*/*z* range of 100–1400 for MS scans and 60–1400 for MS/MS scans, with an acquisition rate of 1 spectrum/s for both modes. The ion source was operated under the following conditions: capillary voltage, 3000 V; nozzle voltage, 1000 V; fragmentor voltage, 110 V; skimmer voltage, 65 V; and octopole RF peak voltage, 750 V. Nitrogen was employed as both the drying and sheath gas at a flow rate of 12 L/min. The drying and sheath gas temperatures were maintained at 250 °C and 300 °C, respectively, while the nebulizer pressure was set to 35 psig. Data acquisition and processing were performed using MassHunter software, version 10.0. Compound identification was based on a combination of UV spectral characteristics, retention times, and interpretation of the observed fragmentation patterns. The obtained data were compared with reference information from established mass spectral databases, including MassBank [69] and PubChem [70], as well as relevant scientific literature. The detected compounds were subsequently annotated in accordance with the identification confidence levels recommended by the Metabolomics Standards Initiative (MSI) [71].

### 4.4. Assessment of Biological Activity

#### 4.4.1. Trolox Equivalet Antioxidant Capacity Assay (TEAC)

The ABTS^•+^ radical cation-scavenging activity of the samples was determined according to the method of Re et al. [72], with minor modifications. To generate the ABTS^•+^ radical cation, 7 mM ABTS and 2.5 mM K_2_S_2_O_8_ were dissolved in 10 mL of ultrapure water and incubated in the dark at room temperature for 16 h. Before analysis, the resulting ABTS^•+^ solution was diluted with absolute ethanol to obtain an absorbance of 0.7–0.8 at 734 nm. Sample solutions were prepared in methanol at a concentration of 1 mg/mL, while Trolox standard solutions were prepared at concentrations of 3.0, 2.0, 1.0, 0.5, 0.25, and 0.125 mM. For the assay, 10 µL of each sample solution was mixed with 990 µL of the ABTS^•+^ working solution. For the control, 10 µL of methanol was added instead of the sample or Trolox solution. After incubation for 30 min, the decrease in absorbance was measured at 734 nm and used to construct the Trolox calibration curve. The ABTS^•+^ scavenging activity was expressed as TEAC, mM and calculated using the linear regression equation obtained from the Trolox standards: *y* = 23.075*x* − 2.5945 (R^2^ = 0.9924; Figure S30).

#### 4.4.2. Total Antioxidant Capacity (TAC)—Phosphomolybden Assay

The antioxidant capacity of the samples was assessed using the phosphomolybdenum assay following the procedure reported by Zengin et al. [73]. Sample solutions were prepared at a concentration of 1 mg/mL and mixed with freshly prepared phosphomolybdenum reagent containing 0.6 M sulfuric acid, 28 mM sodium phosphate, and 4 mM ammonium molybdate. The reaction mixtures were incubated at 95 °C for 90 min and subsequently allowed to cool to room temperature. Absorbance was then recorded at 695 nm. The TAC was determined using an ascorbic acid calibration curve (Figure S31) and expressed as milligrams of ascorbic acid equivalents per gram of extract (mg AsAE/g_extr_).

#### 4.4.3. Ferric Reducing Antioxidant Power (FRAP)

Ferric ion-reducing capacity was determined using a modified FRAP assay based on the method reported by Wong et al. [74]. The required stock solutions consisted of 300 mM acetate buffer (pH 3.6), 10 mM 2,4,6-tris(2-pyridyl)-S-triazine (TPTZ) prepared in 40 mM HCl, and 20 mM FeCl_3_·6H_2_O. The FRAP working reagent was freshly prepared by combining the solutions in a ratio of 10:1:1 (acetate buffer:TPTZ:FeCl_3_). For the assay, 15 µL of each sample solution prepared in methanol containing 10% dimethyl sulfoxide (DMSO) was mixed with 285 µL of the freshly prepared FRAP reagent. The reaction mixtures were incubated at 37 °C for 30 min in the dark, after which the absorbance was measured at 593 nm. A Trolox calibration curve was used for quantification (Figure S32). The ferric ion-reducing antioxidant capacity was expressed as milligrams of Trolox equivalents per gram of dry extract (mg TE/g_extr_).

#### 4.4.4. Free Radical Scavenging Activity (DPPH Assay)

The hydrogen-atom- and electron-donating capacities of the samples were assessed based on their ability to reduce the purple-colored DPPH radical. The DPPH radical-scavenging activity was determined according to the method of Brand-Williams et al. [75], with minor modifications. A fresh DPPH solution (0.08 mg/mL) was prepared daily in methanol and stored at 4 °C in the dark between measurements. Serial dilutions of the test samples (1.0–0.0078 mg/mL) and reference standards, including gallic acid (0.1–0.00078 mg/mL), ascorbic acid (0.50–0.0039 mg/mL), and Trolox (0.75–0.0058 mg/mL), were prepared in methanol containing 10% DMSO directly in 96-well microtiter plates. Methanol was used as the positive control in the final column of the plate. For the assay, 100 µL of each sample, essential oil, or standard solution was mixed with 100 µL of the DPPH solution using an Eppendorf Research^®^ plus multichannel pipette (Eppendorf, Hamburg, Germany). The plates were incubated in the dark for 30 min, after which the decrease in absorbance was measured at 517 nm. Control wells contained 100 µL of methanol instead of the test solution and were mixed with 100 µL of DPPH solution. All measurements were performed in triplicate. The DPPH radical-scavenging activity was expressed as a percentage of inhibition, calculated using the following equation:

$$\mathrm{Inh}\%\;=\;(\frac{{Abs}_{control}-{Abs}_{sample}}{{Abs}_{control}})\times 100$$

IC_50_ values were estimated from plots of DPPH radical-scavenging activity (%) as a function of the corresponding sample concentrations.

#### 4.4.5. Antimicrobial Activity

The antimicrobial activity of the extract was assessed by the broth microdilution technique following the guidelines of the European Committee on Antimicrobial Susceptibility Testing (EUCAST) [76]. The antibacterial properties were evaluated against a panel of Gram-positive bacteria, including *Staphylococcus aureus* ATCC 25923, *S. aureus* ATCC BA1707, *Staphylococcus epidermidis* ATCC 12228, *Micrococcus luteus* ATCC 10240, *Bacillus cereus* ATCC 10876, and *Enterococcus faecalis* ATCC 29212, as well as Gram-negative bacteria, namely *Salmonella* Typhimurium ATCC 14028, *Escherichia coli* ATCC 25922, *Proteus mirabilis* ATCC 12453, *Klebsiella pneumoniae* ATCC 13883, and *Pseudomonas aeruginosa* ATCC 9027. Mueller–Hinton (MH) broth was used as the culture medium for bacterial strains. Antifungal activity was determined against *Candida glabrata* ATCC 90030, *Candida albicans* ATCC 102231, and *Candida parapsilosis* ATCC 22019 using MH broth supplemented with 2% glucose. Prior to the assay, the extract was dissolved in DMSO, diluted with the corresponding culture medium, and subjected to serial two-fold dilutions. The resulting dilutions were dispensed into sterile 96-well microtiter plates (Nunc, Roskilde, Denmark) to obtain final extract concentrations ranging from 20 to 0.156 mg/mL. Fresh microbial cultures were suspended in sterile 0.85% NaCl and standardized to a turbidity equivalent to 0.5 McFarland. The standardized inocula were subsequently added to the wells to achieve final concentrations of 5 × 10^5^ CFU/mL for bacteria and 5 × 10^4^ CFU/mL for yeasts. The plates were incubated at 35 °C for 18–20 h. The MIC was established as the lowest extract concentration that prevented visible microbial growth. Growth inhibition was assessed visually and confirmed spectrophotometrically by measuring absorbance at 600 nm using a BioTek ELx800 microplate reader (BioTek Instruments, Inc., Winooski, VT, USA). To determine the MBC/MFC, 5 μL aliquots from each well were transferred onto extract-free MH agar plates and incubated at 35 °C for 24 h. The lowest concentration resulting in complete inhibition of microbial growth was recorded as the MBC or MFC, respectively. All experiments were conducted in triplicate, and the highest value obtained was reported. The assay included appropriate controls: DMSO at a final concentration of 1% (*v*/*v*), an inoculum control without extract, and an extract control without microbial inoculum.

#### 4.4.6. α-Amylase Enzyme Inhibition Assay

The inhibitory effect of the samples on α-amylase activity was evaluated using the Caraway–Somogyi iodine/potassium iodide (I_2_/KI) colorimetric method [77,78], with minor modifications. A 0.05% substrate solution was prepared by dissolving 10 mg of soluble potato starch in 20 mL of ultrapure water, followed by boiling for 10 min and cooling to room temperature prior to use. Acarbose (0.01–0.1 mg/mL prepared in buffer) was used as the reference inhibitor. For the assay, 20 mM sodium phosphate buffer (pH 6.9) was dispensed into 96-well flat-bottom microplates using a multichannel automatic pipette (Eppendorf, Germany). Subsequently, 25 μL of the sample solution and 50 μL of α-amylase solution (0.8 U/mL in buffer) were added, and the reaction mixtures were incubated at 37 °C for 10 min. Following pre-incubation, 50 μL of the starch substrate solution was added, and the mixtures were further incubated at 37 °C for 10 min. The enzymatic reaction was terminated by adding 25 μL of 1 M HCl, followed by the addition of 100 μL of the I_2_/KI reagent. Sample blanks were prepared using all assay components except the enzyme, which was replaced with 50 μL of buffer. Control wells contained all reaction components with 25 μL of the solvent used to prepare the samples instead of the sample solution and represented 100% enzyme activity. Absorbance was measured at 630 nm for both samples and blanks. The percentage inhibition of α-amylase activity (Inh%) was calculated using the following equation:

$$\mathrm{Inh}\%\;=\;\left[\frac{\left({Abs}_{control}\;-\;{Abs}_{control\;blank}\right)\;-\;\left({Abs}_{sample}\;-{\;Abs}_{sample\;blank}\right)}{{Abs}_{control\;}-\;{Abs}_{control\;blank}}\right]\times 100$$

### 4.5. Statistical Analysis

Statistical analyses were conducted using SigmaPlot software (version 12.0). Data obtained from the inhibition assays are presented as the mean ± standard error of the mean (SEM). The IC_50_ values for the DPPH radical-scavenging assay were estimated by nonlinear regression analysis.

## 5. Conclusions

The present study provides the first comprehensive phytochemical characterization and biological evaluation of *F. varia* root extract. HPLC–ESI–QTOF–MS/MS analysis revealed a chemically diverse metabolite profile dominated by sesquiterpene coumarins, together with phenolic acids, coumarins, organic acids, sesquiterpene esters, and prenylated aromatic compounds.

The biological evaluation demonstrated that the extract possesses moderate antioxidant activity, measurable α-amylase inhibitory potential, and broad-spectrum antimicrobial activity against Gram-positive and Gram-negative bacteria as well as pathogenic yeasts, with the highest susceptibility observed for *C. glabrata*. The observed biological effects are likely associated with the combined action of the identified phenolic acids and characteristic sesquiterpene coumarins, which have previously been reported to exhibit antioxidant, enzyme inhibitory, antimicrobial, and antifungal properties.

The present study substantially expands the phytochemical knowledge of *F. varia* roots. Several metabolites previously reported from other *Ferula* species were tentatively identified here for the first time in *F. varia* roots, demonstrating that the underground organs of this species represent a considerably richer source of specialized metabolites than previously recognized.

Overall, the results indicate that *F. varia* roots represent a promising source of biologically active secondary metabolites and support their potential as a valuable raw material for the development of phytopharmaceuticals and other plant-derived bioactive products.

## Figures and Tables

**Figure 1:**
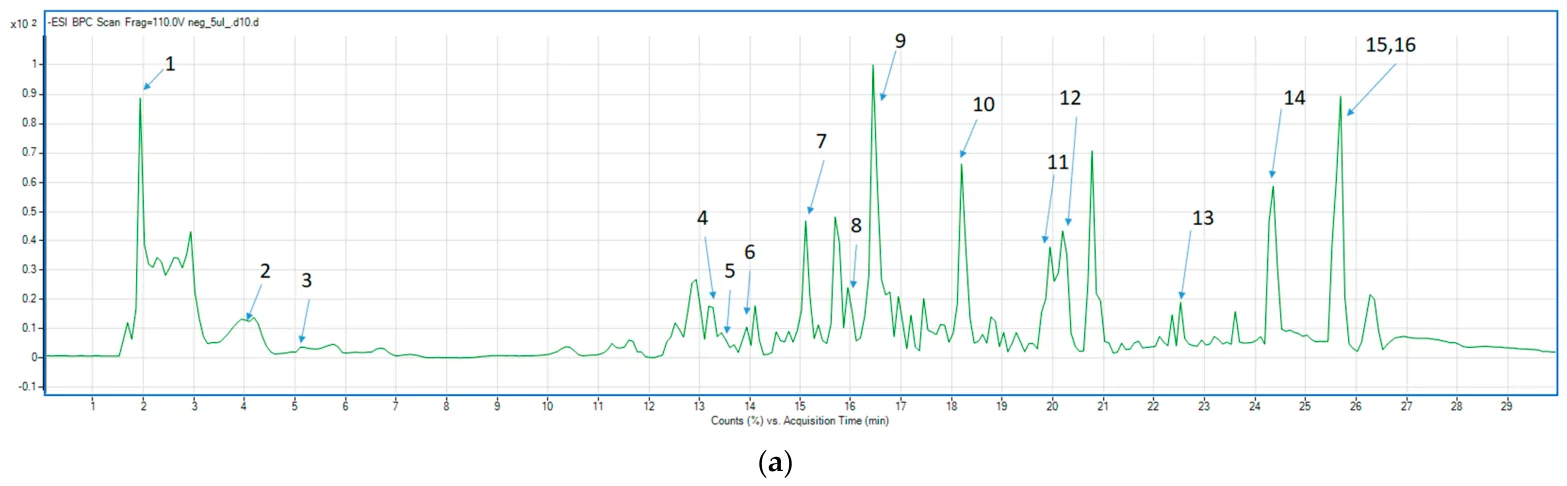

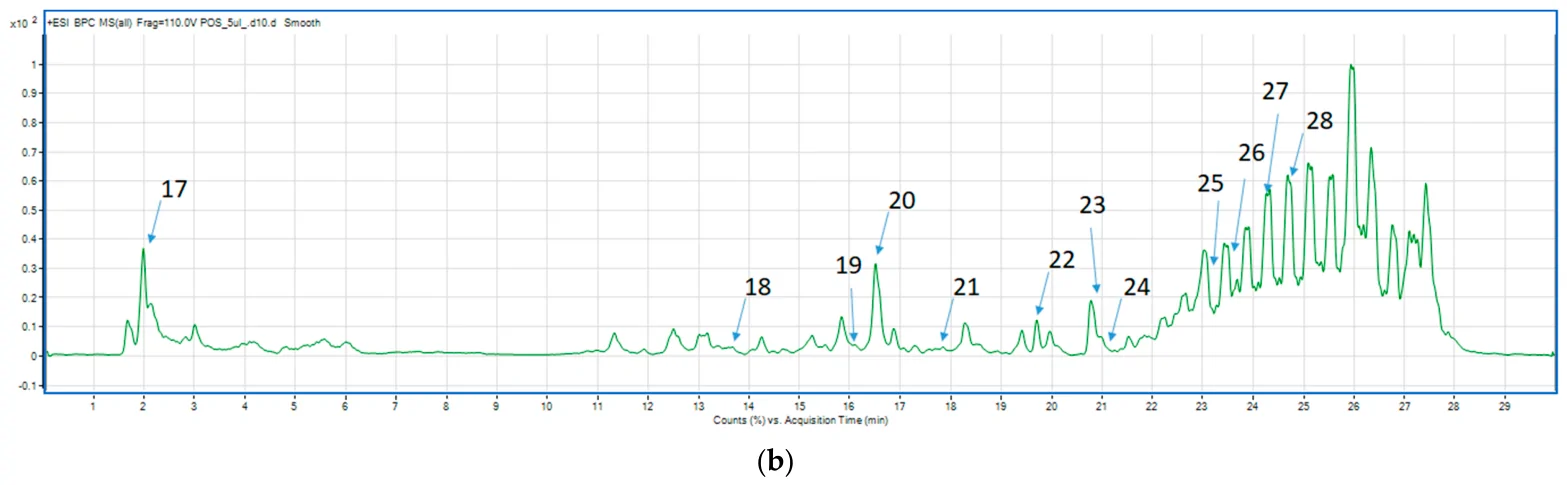
Base peak chromatogram (BPC) of the *F. varia* root extract (HPLC-ESI-QTOF-MS/MS): (**a**) negative ionization mode and (**b**) positive ionization mode.

**Table 1:** Total phenolic and flavonoid contents of *F. varia* root extract.

Sample Name	TFC mg RuE/g_extr_	TPC mg GAE/g_extr_
*F. varia* roots extract	1.42 ± 0.04	30.06 ± 3.00

RuE—rutin equivalent; GAE—gallic acid equivalent.

**Table 2:** HPLC-ESI-QTOF-MS/MS analysis of the *F. varia* root extract in negative and positive ionization mode.

№	RT (min)	Formula	Theoretical (*m*/*z*)	Experimental (*m*/*z*)	∆ ppm	DBE	MS/MS Fragments	Tentative Identification	Ref.	MSI Level
Negative mode										
1	1.930	C_12_H_8_O_4_	215.035	215.0346	1.77	9	89.0264 113.0259	Bergapten	[22]	2
2	3.964	C_6_H_8_O_7_	191.0197	191.0209	−6.11	3	87.0094 111.0092	Citric acid	[23]	2
3	5.065	C_4_H_6_O_4_	117.0193	117.0191	1.97	2	73.0207 98.9989	Succinic acid	[24]	2
4	13.570	C_10_H_10_O_4_	193.0506	193.0521	−7.56	6	74.9848 105.0301 136.9824	Ferulic acid	[25]	2
5	13.920	C_24_H_26_O_5_	393.1707	393.1733	−6.48	12	89.0240 161.0446 240.9991 347.1679	Conferdione isomer	[26]	2
6	15.154	C_27_H_26_O_4_	413.1758	413.1771	−3.06	15	101.0281 235.1248 367.1700	Olgoferin	[27]	2
7	16.155	C_24_H_28_O_5_	395.1864	395.1886	−5.56	11	89.0244 179.0541 349.1843	Karatavic acid	[26]	2
8	16.405	C_16_H_18_O_9_	353.0878	353.0893	−4.22	8	161.0288 191.0564	Chlorogenic acid	[28]	2
9	18.223	C_20_H_20_O_5_	339.1238	339.1248	−2.95	11	89.0466 159.0527 249.0714	Flavaprenin	[26]	2
10	19.841	C_9_H_6_O_3_	161.0244	161.0243	0.73	7	105.0347 133.0293	Umbelliferone	[24]	2
11	20.275	C_10_H_10_O_3_	177.0557	177.0564	−3.83	6	89.0422	3-Methoxycinnamic acid	[26]	2
12	22.576	C_24_H_28_O_4_	379.1915	379.1908	1.8	11	84.0087 174.0324 242.9493 311.1890	Conferone	[26]	2
13	24.327	C_22_H_30_O_5_	373.202	373.2016	1.2	8	153.0197	Ferugin	[29]	2
14	25.545	C_22_H_30_O_3_	341.2122	341.2103	5.61	8	106.0429 204.0777 297.2206	Teferdin	[23]	2
15	25.678	C_22_H_30_O_4_	357.2071	357.2058	3.72	8	93.0340 137.0223 219.1734	Ferutinin	[30]	2
Positive mode										
16	2.055	C_11_H_6_O_5_	219.0288	219.0265	10.55	9	60.0834 104.1057	5,8-dihydroxypsoralen	[31]	2
17	13.595	C_12_H_8_O_4_	217.0495	217.0495	0.16	9	90.0537 111.0044 160.9871	Xanthotoxin	[32]	2
18	16.030	C_11_H_12_O_3_	193.0859	193.0841	9.48	6	105.0692 133.0640 161.0591	Myristicin	[33]	2
19	16.563	C_16_H_18_O_9_	355.1024	355.1026	−0.68	8	163.0376	Chlorogenic acid	[28]	2
20	17.914	C_19_H_22_O_5_	331.154	331.1507	10	9	85.0265 169.0830 287.1061	Ferusinkin A	[26]	2
21	19.766	C_9_H_6_O_3_	163.039	163.0383	4.14	7	107.0485	Umbelliferone	[24]	2
22	20.950	C_24_H_28_O_4_	381.206	381.2063	−0.69	11	95.0849 201.1629 279.0982	Badrakemone	[26]	2
23	21.100	C_24_H_30_O_5_	399.2166	399.2169	−0.75	10	71.0497 133.0997 163.0385 293.1216	Galbanic acid	[34]	2
24	23.251	C_26_H_30_O_5_	423.2166	423.2141	5.92	12	109.0983 185.0199 261.1821	Omega-acetoxyferprenin	[35]	2
25	23.702	C_26_H_34_O_6_	443.2428	443.247	−9.46	10	109.0999 203.1788 383.2225	Karatavicin	[26]	2
26	24.119	C_24_H_28_O_4_	381.206	381.2056	1.15	11	105.0687 163.0382 201.1631	Farnesiferone B	[31]	2
27	24.786	C_24_H_30_O_4_	383.2217	383.2221	−1.08	10	95.0840 163.0384 203.1789 269.1188 318.2409	Badrakemin	[26]	2

RT—retention time; DBE—number of double bonds and rings; ∆ ppm—error of *m*/*z* measurement.

**Table 3:** Antioxidant activity of *F. varia* root extract (mean ± SD).

Method	Result
TEAC, mM	0.10 ± 0.00
TAC, mg AsAE/g_extr_	110.31 ± 4.94
FRAP, mgTE/g_extr_	21.95 ± 0.00
DPPH IC_50_, mg/mL	>1.0

AsA: ascorbic acid equivalent; TE: Trolox equivalent.

**Table 4:** α-Amylase enzyme inhibitory potential of the extract (mean ± SD).

Antidiabetic Potential	Result
α-Amylase Enzyme Inhibition, mg AcrE/g_extr_	6.06 ± 0.55

AcrE: acarbose equivalent.

**Table 5:** Antimicrobial activity of investigated *F. varia* extract (mg/mL).

Microorganisms	Gram-Positive Bacteria		Microorganisms	Gram-Negative Bacteria		Microorganisms	Yeasts	
	MIC	MBC		MIC	MBC		MIC	MFC
*Staphylococcus aureus* ATCC 25923	20	20	*Salmonella* Typhimurium ATCC 14028	10	>20	*Candida glabrata* ATCC 90030	2.5	5
*Staphylococcus aureus* ATCC BA1707	20	20	*Escherichia coli* ATCC 25922	10	20	*Candida albicans* ATCC 102231	5	5
*Staphylococcus epidermidis* ATCC 12228	20	20	*Proteus mirabilis* ATCC 12453	20	20	*Candida parapsilosis* ATCC 22019	5	5
*Micrococcus luteus* ATCC 10240	5	10	*Klebsiella pneumoniae* ATCC 13883	10	>20			
*Bacillus cereus* ATCC 10876	10	>20	*Pseudomonas aeruginosa* ATCC 9027	10	20			
*Enterococcus faecalis* ATCC 29212	20	>20						

**Table 6:** Reference antibiotics activity against tested microbial strains (mg/L).

Microorganisms	Gram-Positive Bacteria	Microorganisms	Gram-Negative Bacteria	Microorganisms	Yeasts
	MIC (Vancomycin)		MIC (Ciprofloxacin)		MIC (Nystatin)
*S. aureus* ATCC 25923	0.98	*S.* Typhimurium ATCC 14028	0.061	*C. glabrata* ATCC 90030	0.24
*S. aureus* *ATCC BAA1707*	0.98	*E. coli* ATCC 25922	0.015	*C. albicans* ATCC 102231	0.48
*S. epidermidis* ATCC 12228	0.98	*P. mirabilis* ATCC 12453	0.030	*C. parapsilosis* ATCC 22019	0.24
*M. luteus* ATCC 10240	0.12	*K. pneumoniae* ATCC 13883	0.122		
*B. cereus* ATCC 10876	0.98	*P. aeruginosa* ATCC 9027	0.488		
*E. faecalis* ATCC 29212	1.95				

## Data Availability

The original contributions presented in this study are included in the article/Supplementary Materials. Further inquiries can be directed to the corresponding authors.

## Supplementary Materials

The following supporting information can be downloaded at: https://www.mdpi.com/article/10.3390/molecules31172990/s1, Figure S1: MS/MS fragmentation spectrum of bergapten in negative mode; Figure S2: MS/MS fragmentation spectrum of citric acid in negative mode; Figure S3: MS/MS fragmentation spectrum of succinic acid in negative mode; Figure S4: MS/MS fragmentation spectrum of ferulic acid in negative mode; Figure S5: MS/MS fragmentation spectrum of conferdione isomer in negative mode; Figure S6: MS/MS fragmentation spectrum of olgoferin in negative mode; Figure S7: MS/MS fragmentation spectrum of karatavic acid in negative mode, Figure S8: MS/MS fragmentation spectrum of chlorogenic acid in negative mode; Figure S9: MS/MS fragmentation spectrum of flavaprenin in negative mode; Figure S10: MS/MS fragmentation spectrum of umbelliferone in negative mode; Figure S11: MS/MS fragmentation spectrum of 3-methoxycinnamic acid in negative mode; Figure S12: MS/MS fragmentation spectrum of conferone in negative mode; Figure S13: MS/MS fragmentation spectrum of ferugin in negative mode; Figure S14: MS/MS fragmentation spectrum of teferdin in negative mode; Figure S15: MS/MS fragmentation spectrum of ferutinin in negative mode; Figure S16: MS/MS fragmentation spectrum of 5,8-dihydroxypsoralen in positive mode; Figure S17: MS/MS fragmentation spectrum of xanthotoxin in positive mode; Figure S18: MS/MS fragmentation spectrum of myristicin in positive mode; Figure S19: MS/MS fragmentation spectrum of chlorogenic acid in positive mode; Figure S20: MS/MS fragmentation spectrum of ferusinkin A in positive mode; Figure S21: MS/MS fragmentation spectrum of umbelliferone in positive mode; Figure S22: MS/MS fragmentation spectrum of badrakemone in positive mode; Figure S23: MS/MS fragmentation spectrum of galbanic acid in positive mode; Figure S24: MS/MS fragmentation spectrum of omega-acetoxyferprenin in positive mode; Figure S25: MS/MS fragmentation spectrum of karatavicin in positive mode; Figure S26: MS/MS fragmentation spectrum of farnesiferone B in positive mode; Figure S27: MS/MS fragmentation spectrum of badrakemin in positive mode; Figure S28: Gallic acid calibration curve; Figure S29: Rutin calibration curve; Figure S30: Calibration curve for Trolox; Figure S31: Calibration curve obtained with ascorbic acid; Figure S32: Calibration curve for Trolox.

## Abbreviations

The following abbreviations are used in this manuscript:

AcrE	Acarbose equivalent
AsA	Ascorbic acid equivalent
BPC	Base peak chromatogram
DBE	Number of double bonds
DMSO	Dimethyl sulfoxide
DPPH	2,2-diphenyl-1-picrylhydrazyl
EUCAST	European Committee on Antimicrobial Susceptibility Testing
FRAP	Ferric reducing antioxidant power
GAE	Gallic acid equivalent
HPLC–QTOF–ESI-MS/MS	High-performance liquid chromatography coupled with mass spectrometry
MBC	Minimum bactericidal concentration
MFC	Minimum fungicidal concentration
MH	Mueller-Hinton
MIC	Minimum inhibitory concentration
MSI	Metabolomics Standards Initiative
RT	Retention time
RuE	Rutin equivalent
SEM	Standard error of the mean
TAC	Total antioxidant capacity
TE	Trolox equivalent
TEAC	Trolox equivalent antioxidant capacity
TFC	Total flavonoid content
TPC	Total phenolic content
TPTZ	2,4,6-tris(2-pyridyl)-S-triazine
