# Supplementary material for: Ferula varia Trautv. Root Extract: Extraction, Phytochemical Characterization and Evaluation of Biological Activity
Source: Molecules. 2026 Aug 26;31(17):2990. doi: 10.3390/molecules31172990 (PMC13567091; doi:10.3390/molecules31172990)
Supplement: Supplementary file 1 [file molecules-31-02990-s001.zip › molecules-4523606-supplementary.pdf]

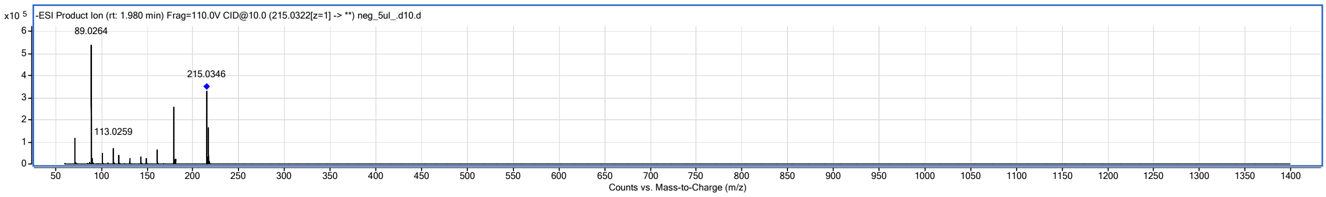

Figure S1. MS/MS fragmentation spectrum of bergapten in negative mode.

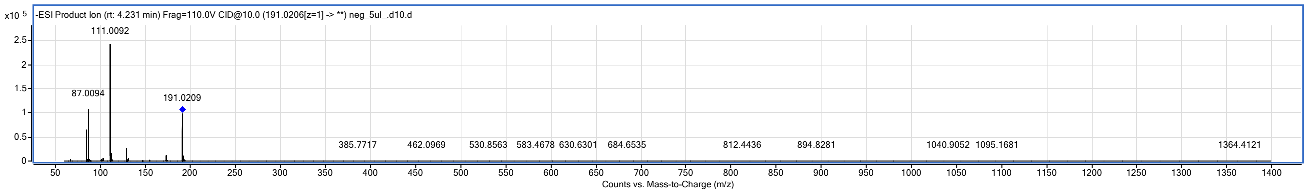

Figure S2. MS/MS fragmentation spectrum of citric acid in negative mode.

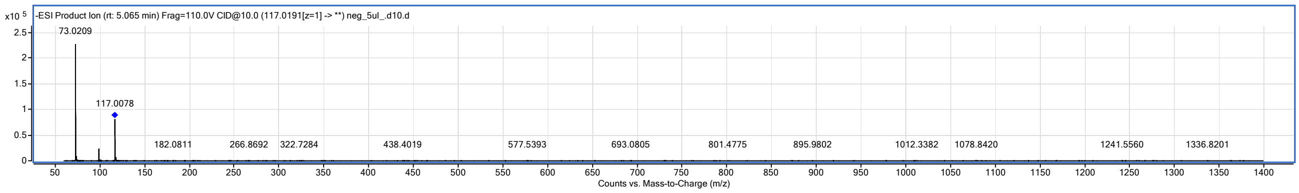

Figure S3. MS/MS fragmentation spectrum of succinic acid in negative mode.

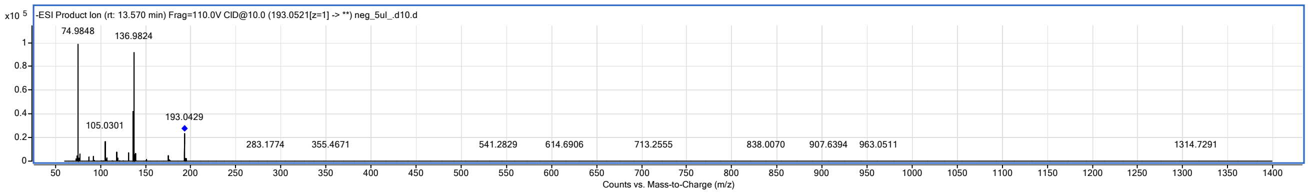

Figure S4. MS/MS fragmentation spectrum of ferulic acid in negative mode.

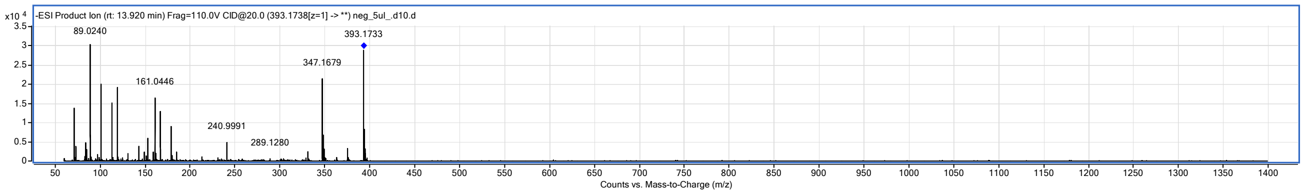

Figure S5. MS/MS fragmentation spectrum of conferdione isomer in negative mode.

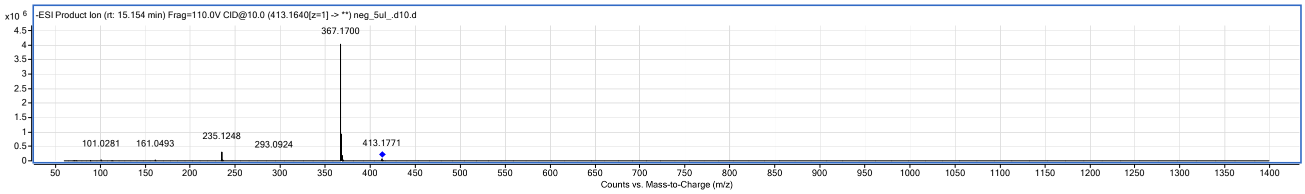

Figure S6. MS/MS fragmentation spectrum of olgoferin in negative mode.

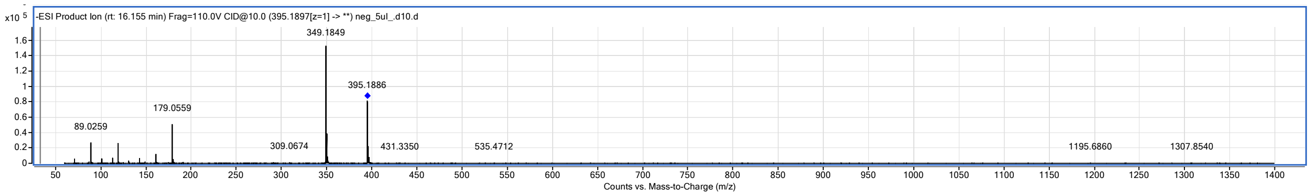

Figure S7. MS/MS fragmentation spectrum of karatavic acid in negative mode.

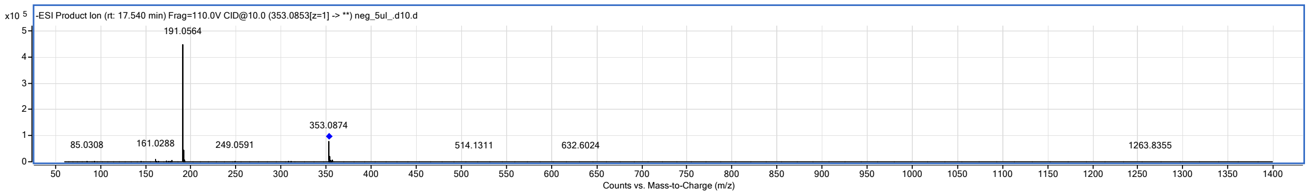

Figure S8. MS/MS fragmentation spectrum of chlorogenic acid in negative mode.

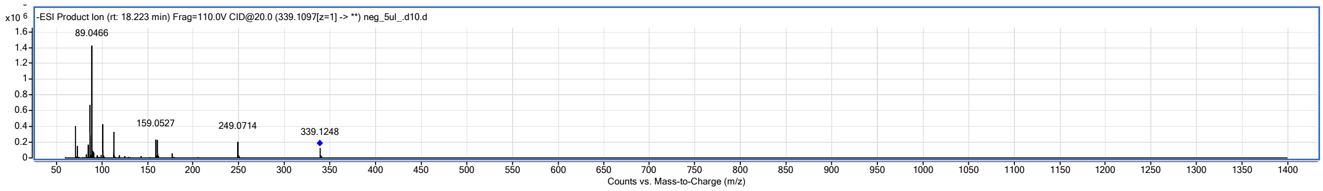

Figure S9. MS/MS fragmentation spectrum of flavaprenin in negative mode.

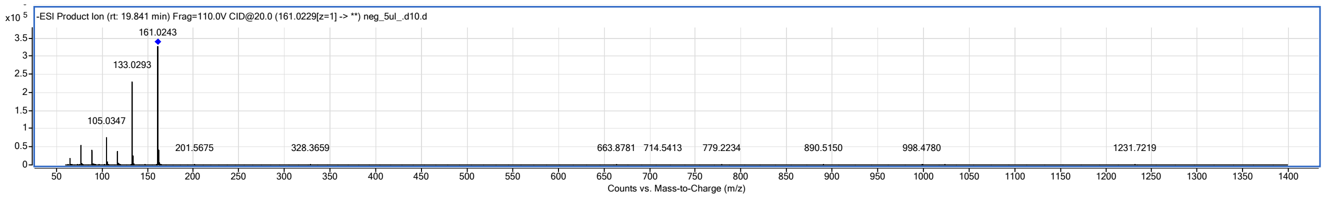

Figure S10. MS/MS fragmentation spectrum of umbelliferone in negative mode.

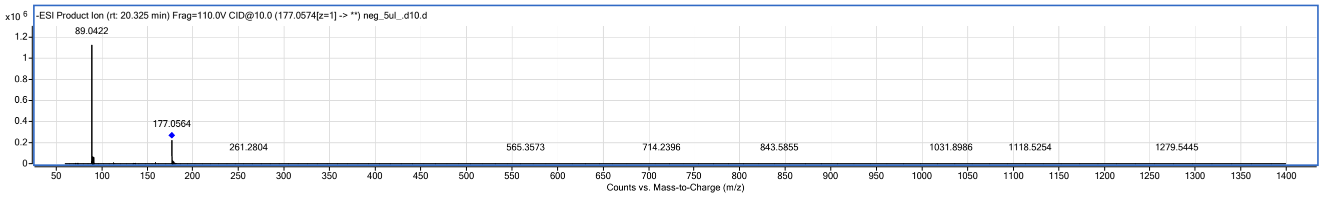

Figure S11. MS/MS fragmentation spectrum of 3-methoxycinnamic acid in negative mode.

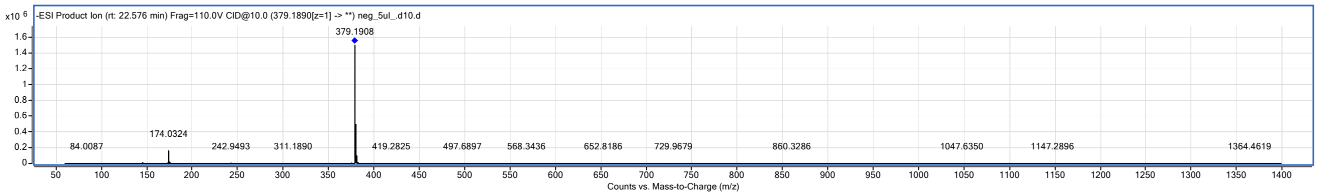

Figure S12. MS/MS fragmentation spectrum of conferone in negative mode.

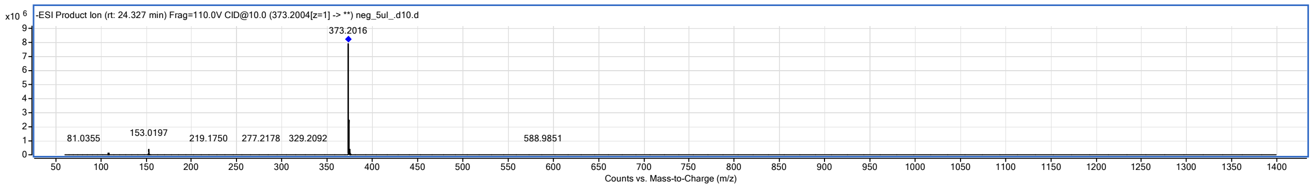

Figure S13. MS/MS fragmentation spectrum of ferugin in negative mode.

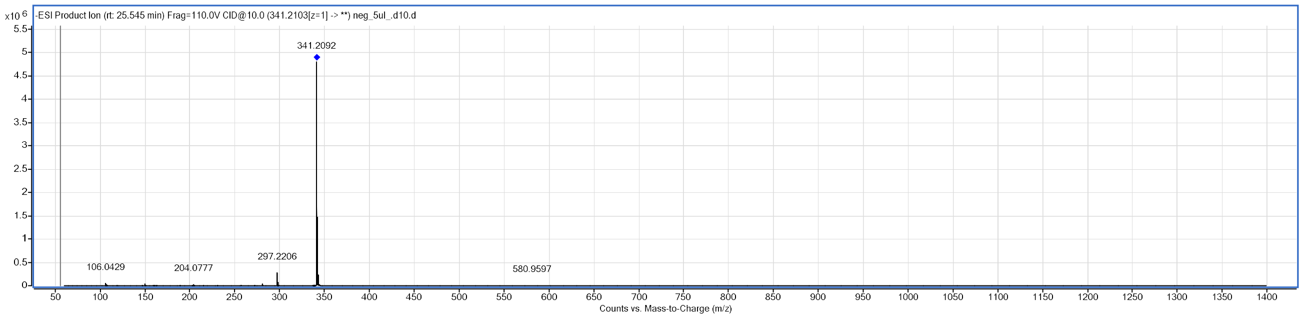

Figure S14. MS/MS fragmentation spectrum of teferdin in negative mode.

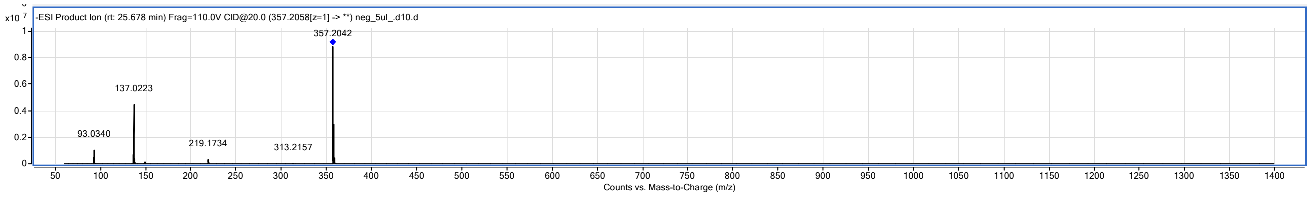

Figure S15. MS/MS fragmentation spectrum of ferutinin in negative mode.

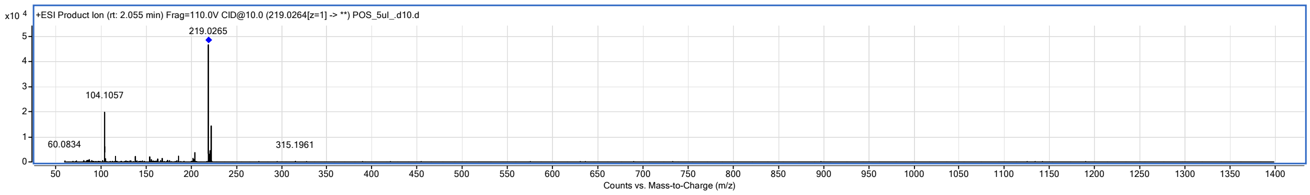

Figure S16. MS/MS fragmentation spectrum of 5,8-dihydroxypsoralen in positive mode.

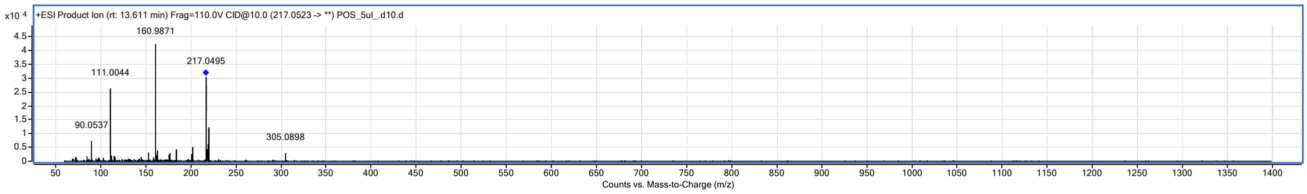

Figure S17. MS/MS fragmentation spectrum of xanthotoxin in positive mode.

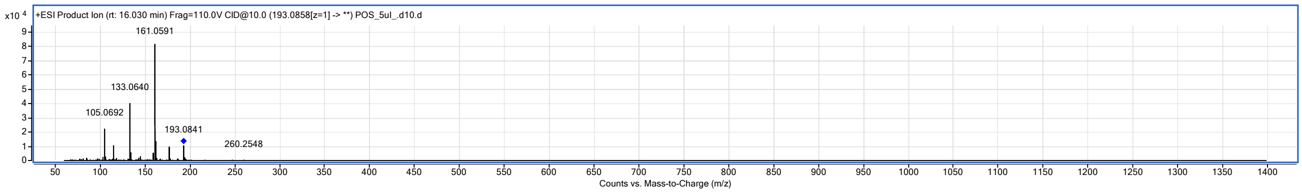

Figure S18. MS/MS fragmentation spectrum of myristicin in positive mode.

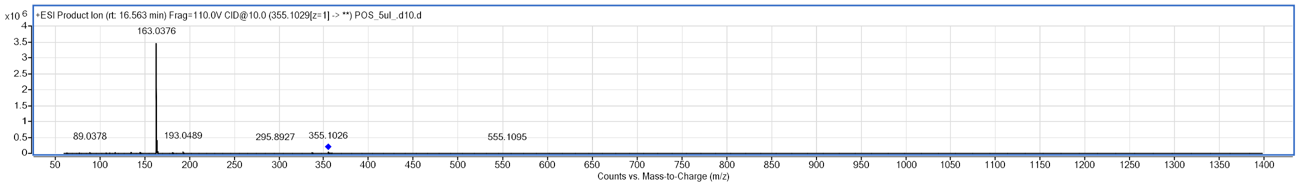

Figure S19. MS/MS fragmentation spectrum of chlorogenic acid in positive mode.

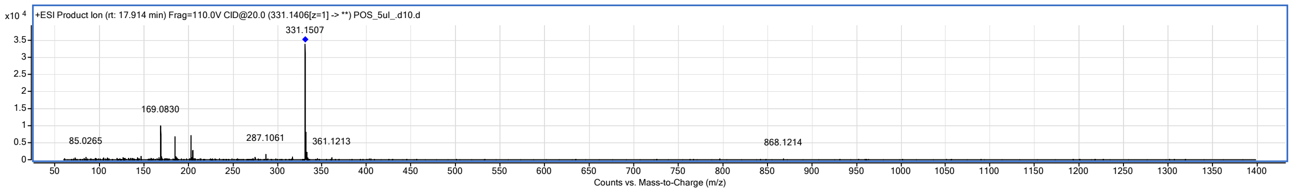

Figure S20. MS/MS fragmentation spectrum of ferusinkin A in positive mode.

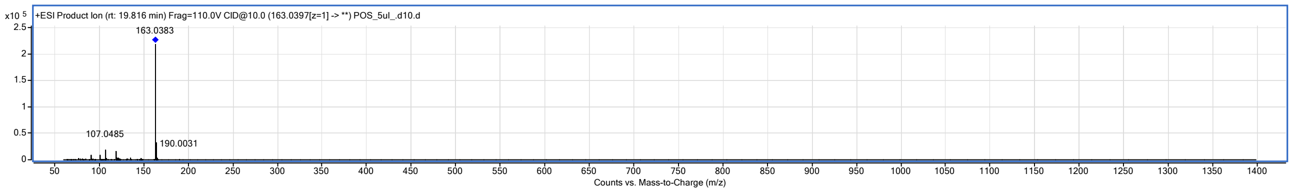

Figure S21. MS/MS fragmentation spectrum of umbelliferone in positive mode.

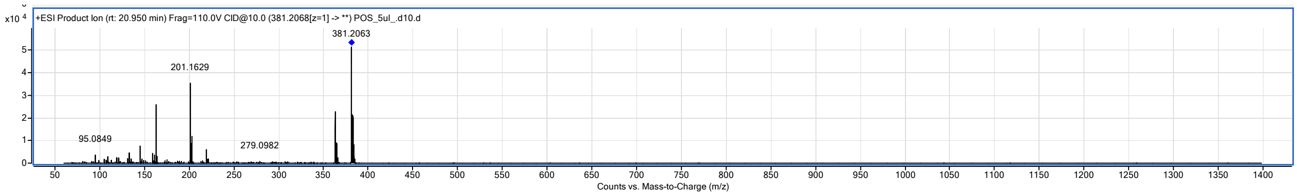

Figure S22. MS/MS fragmentation spectrum of badrakemone in positive mode.

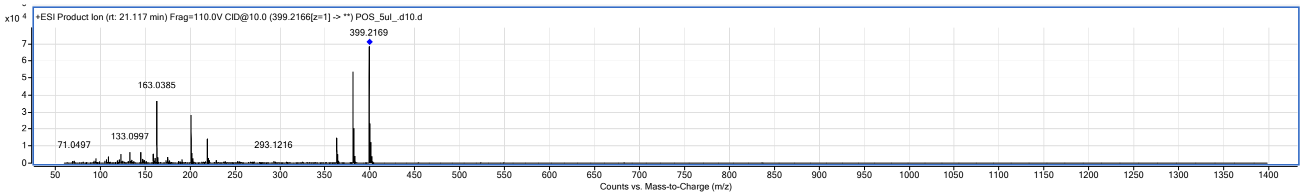

Figure S23. MS/MS fragmentation spectrum of galbanic acid in positive mode.

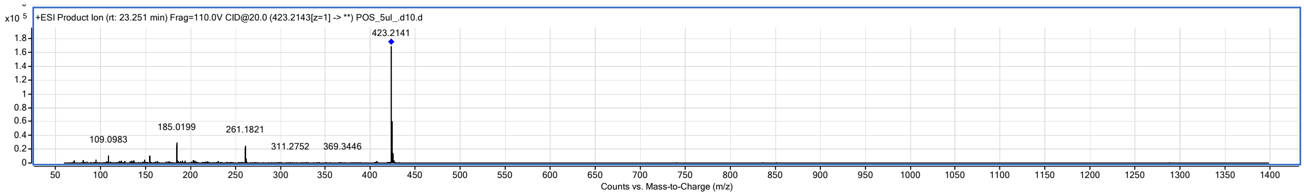

Figure S24. MS/MS fragmentation spectrum of omega-acetoxyferprenin in positive mode.

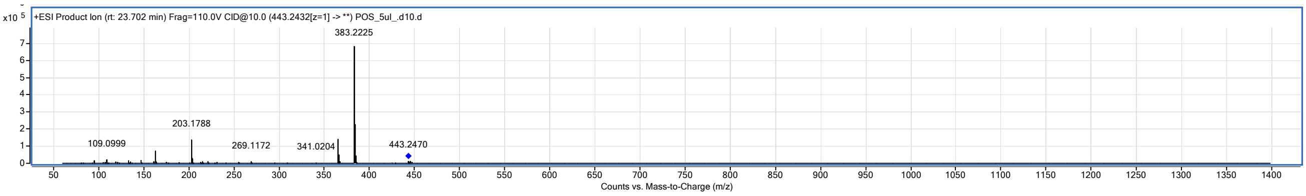

Figure S25. MS/MS fragmentation spectrum of karatavicin in positive mode.

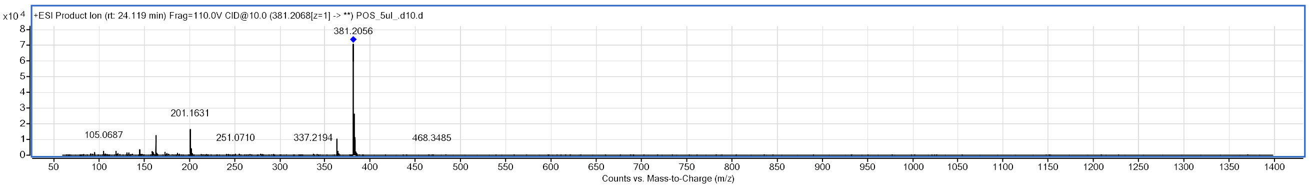

Figure S26. MS/MS fragmentation spectrum of farnesiferone B in positive mode.

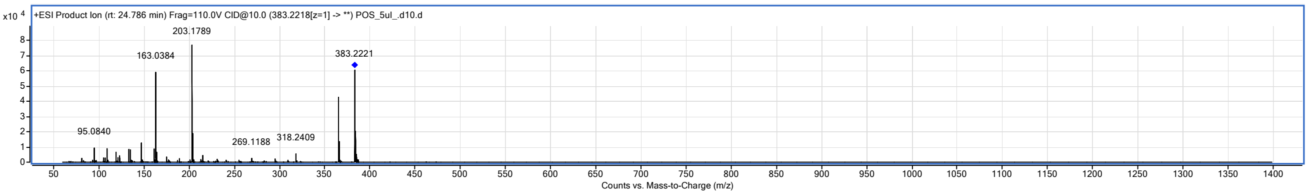

Figure S27. MS/MS fragmentation spectrum of badrakemin in positive mode.

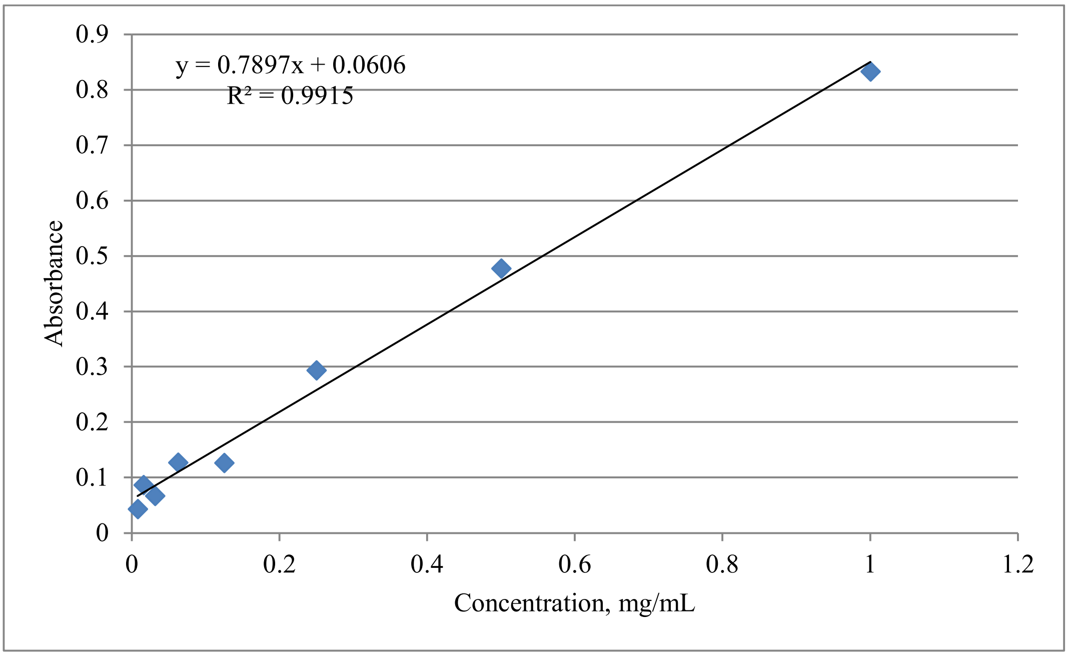

Figure S28. Gallic acid calibration curve.

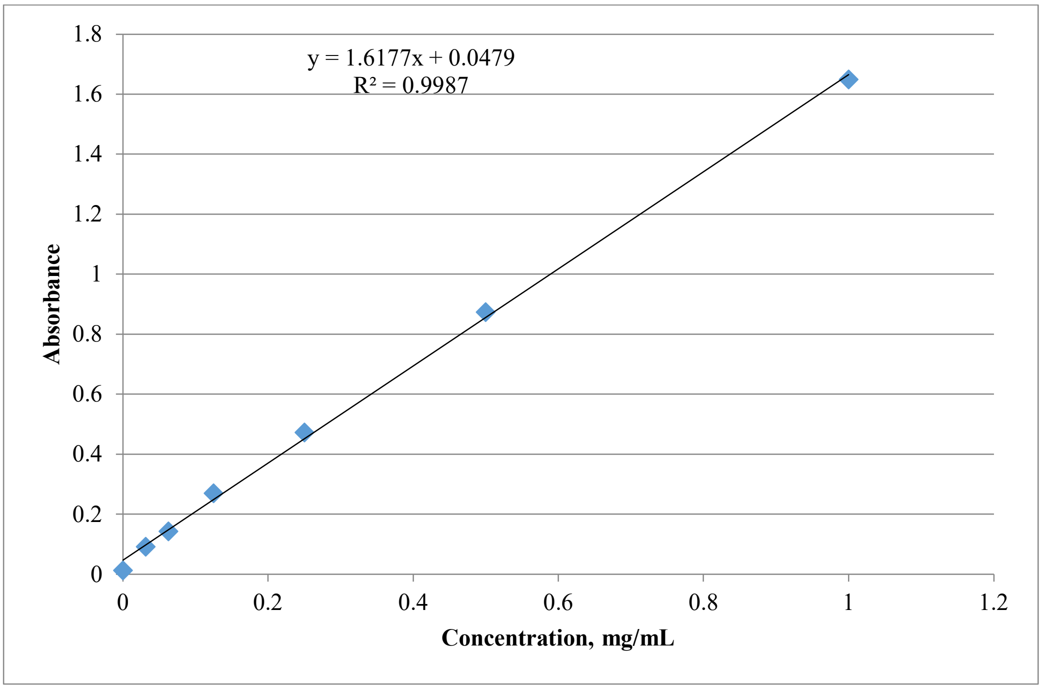

Figure S29. Rutin calibration curve.

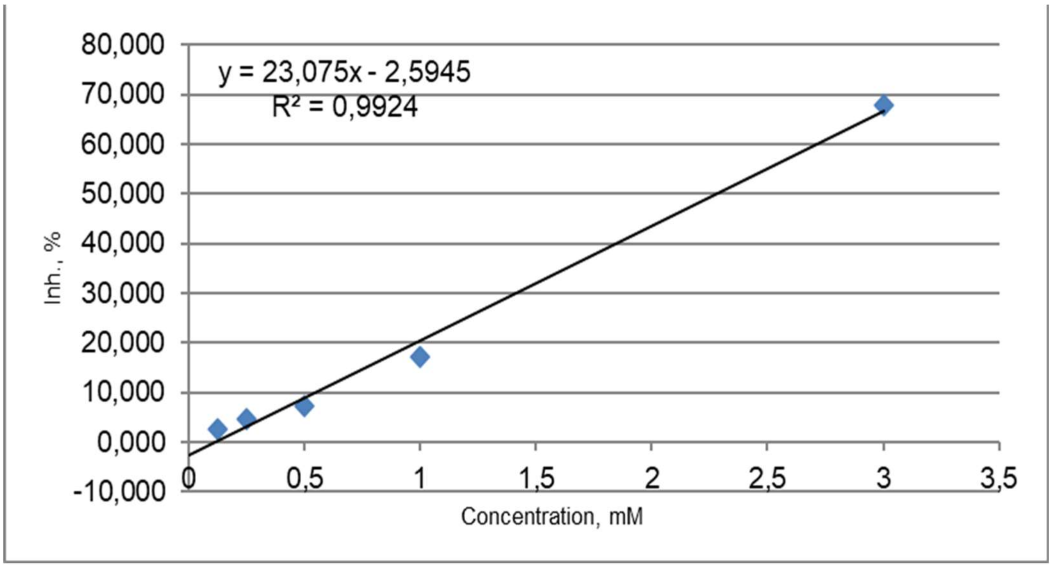

Figure S30. Calibration curve for Trolox.

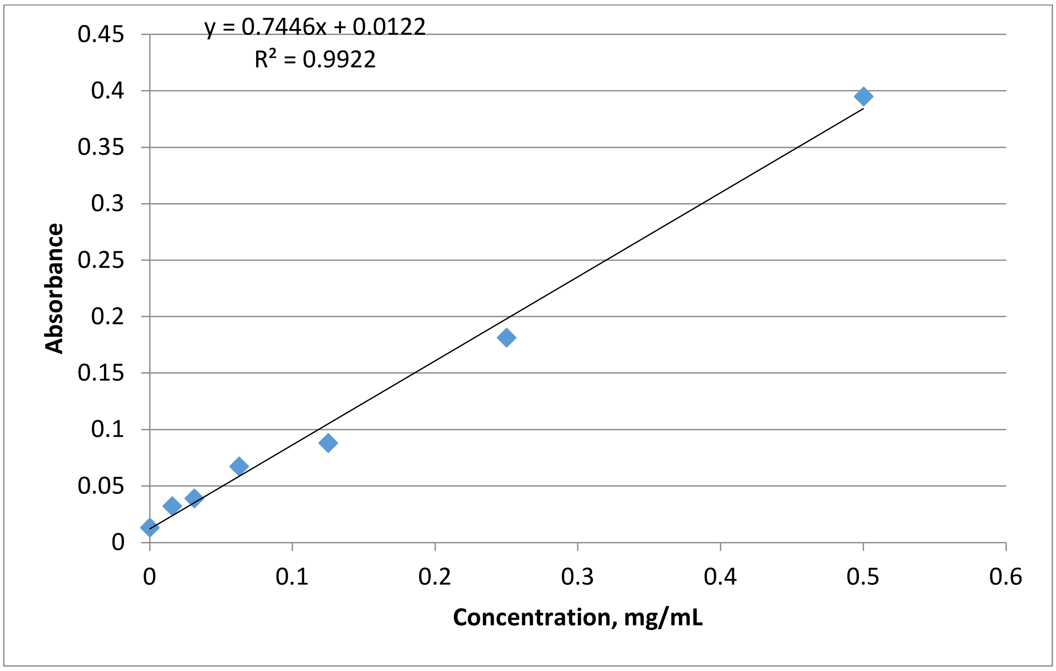

Figure S31. Calibration curve obtained with ascorbic acid.

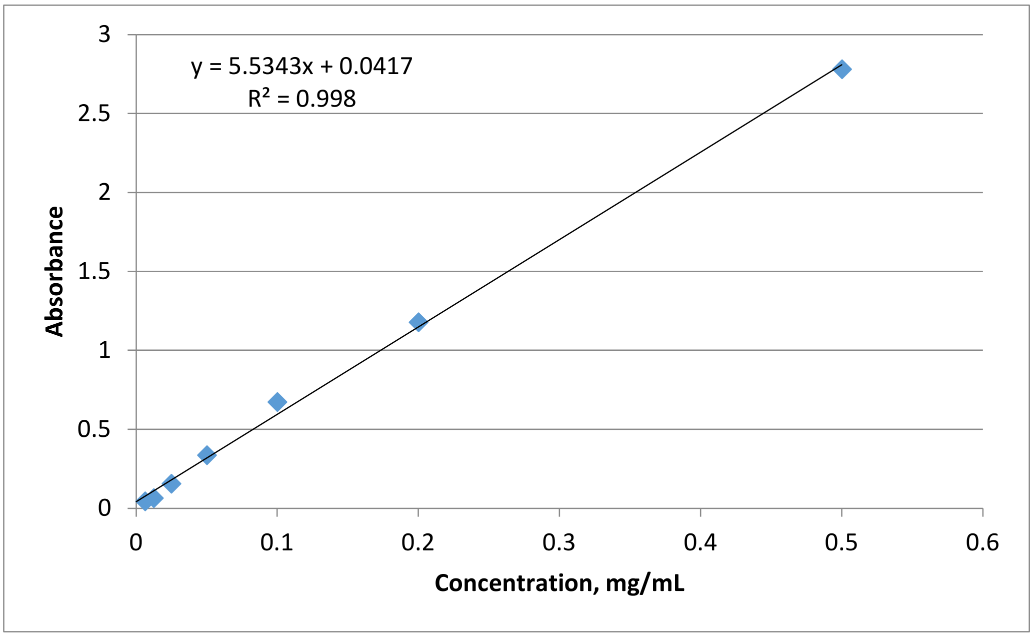

Figure S32. Calibration curve for Trolox.
